# Cordyceps industry in China

**DOI:** 10.1080/21501203.2015.1043967

**Published:** 2015-05-21

**Authors:** Caihong Dong, Suping Guo, Wenfeng Wang, Xingzhong Liu

**Affiliations:** a State Key Laboratory of Mycology, Institute of Microbiology, Chinese Academy of Sciences, No 3 Park 1, Beichen West Road, Chaoyang District, Beijing100101, China; b Bioengineering Laboratory, Shanxi Research Institute for Medicine and Life Science, Taiyuan030006, China; c Research and Development Department, Jiangsu Shenhua Pharmaceutical Co., Ltd., Huaian211600, China

**Keywords:** Chinese cordyceps, cordyceps fungus, cordyceps, industry, Chinese cordyceps–associated fungi

## Abstract

Cordyceps, as a general term, describes a group of ascomycetous fungi growing on arthropods and other related fungi. Some cordyceps have been used in traditional Chinese medicine for centuries and cordyceps-derived products are currently a big industry in China. A number of medicinal and health products have been developed and extensively commercialized from natural Chinese cordyceps, its anamorphic fungus (*Hirsutella sinensis*), and other fungi known as Chinese cordyceps. The lack of a defined classification system for medicinal cordyceps fungi is a source of confusion in the industry and the public, and even among pharmaceutical scientists. This review summarizes the cordyceps fungi currently used in the industry in China with a special reference to clarify Chinese cordyceps and associated fungi. *Cordyceps militaris*, *Cordyceps guangdongensis* and *Isaria cicadae* are well recognized and commercialized cordyceps fungi in China. Except the natural Chinese cordyceps and its anamorphic fungus, *Paecilomyces hepiali*, *Mortierella hepiali*, *Cephalosporium sinensis* and *Clonostachys rosea* isolated from natural Chinese cordyceps are classified as Chinese cordyceps–associated fungi. *P. hepiali* is a cordyceps fungus based on current phylogenetic analysis of Hypocreales, while *M. hepiali* is a fungus in the Zygomycetes and should only be treated as associated fungus of Chinese cordyceps. *C. sinensis* and *C. rosea* belong to the Hypocreales and their relationship to cordyceps fungi should be further studied. The exploitation of the resources of cordyceps fungi and their quality control in the industry should be major topics for future studies. Cooperation between the industry and the research community will enhance the whole cordyceps industry.

## Introduction

Cordyceps is a broad term describing a group of ascomycetous fungi that have carved out a niche as endoparasites primarily of arthropods and also as symbionts of the ascomycete truffle genus *Elaphomyces*. So far, 540 species have been reported as cordyceps (http://www.indexfungorum.org/names/Names.asp, 2 January 2015). The term *Cordyceps* is derived from the Latin words, ‘cord’ and ‘ceps’, respectively meaning ‘club’ and ‘head’ (Bhandari et al. 2010). The Latin word conjunction accurately describes the appearance of these ‘club fungi’, whose stroma and fruit body extend from the mummiﬁed carcasses of insect larvae. The term ‘cordyceps’ (lowercase and without italics) has been proposed to refer any cordyceps fungus–host complex (i.e. 虫草 in Chinese), and the term ‘cordyceps fungus’ (i.e. 虫草菌 in Chinese) should be used when referring only to the fungus that forms cordyceps (Zhang et al. 2012). The term ‘Chinese cordyceps’ (or 冬虫夏草 or 中国虫草 in Chinese) specifically refers to the *Ophiocordyceps sinensis*–ghost moth caterpillar complex, while ‘Chinese cordyceps fungus’ or ‘Chinese caterpillar fungus’ (冬虫夏草菌 in Chinese) refers to the common English names for the fungus (Zhang et al. 2012).

Cordyceps fungi (i.e. *Cordyceps* sensu lato) are divided into four genera: *Cordyceps* sensu stricto (belonging to the family Cordycipitaceae), *Ophiocordyceps* (Ophiocordycipitaceae), *Metacordyceps* (Clavicipitaceae) and *Elaphocordyceps* (Ophiocordycipitaceae) (Sung et al. 2007). *Metacordyceps* has partially to completely immersed perithecia and ascospores disarticulated or not. *Elaphocordyceps* is characterized by fibrous and darkly pigmented stromata, superficial to partially immersed perithecia and disarticulating ascospores, and parasitizes on either cicadas or the truffle genus *Elaphomyces. Ophiocordyceps* is composed of those former *Cordyceps* that give rise to darkly pigmented, fibrous and pliant stromata with superficial to partially immersed perithecia, disarticulating or non-disarticulating ascospores. *Cordyceps* species produce stromata with faint to bright pigmentation on the surface of leaf litter or soil and bear superficial to partially immersed perithecia, giving rise to ascospores that may or may not disarticulate (Sung et al. 2007).

Many natural cordyceps are used in traditional Chinese medicines in China, Japan, Korea and other eastern Asian countries (Zhu et al. 1998). Of the different varieties of cordyceps fungi, those presently being developed for medicinal purposes, health supplements and pharmaceutical drugs in China include but not limited to *O. sinensis* (Berk.) G. H. Sung, J. M. Sung, Hywel-Jones & Spatafora (≡ *Cordyceps sinensis* (Berk.) Sacc.), *Cordyceps militaris* (L.) Fr., *Cordyceps guangdongensis* T.H. Li, Q.Y. Lin & B. Song, and *Isaria cicadae* Miq. Various topics on cordyceps and cordyceps fungi have been reviewed: terminology and nomenclature (Zhang et al. 2012; Shrestha et al. 2014), chemical and pharmacological aspects (Paterson 2008; Zhou et al. 2009; Yue et al. 2013), molecular pathogenesis (St. Leger et al. 2011) and genetics (Zheng et al. 2013). The Chinese cordyceps and associated fungi are the largest fungal industry in China and worldwide. Although *Hirsutella sinensis* X.J. Liu, Y.L. Guo, Y.X. Yu & W. Zeng has been well recognized as the anamorph of *O. sinensis* in the mycological community, more than 20 anamorphic fungi were isolated from natural Chinese cordyceps and reported to be connected with the teleomorph of *O. sinensis* (Jiang and Yao 2003). A number of medicinal and health products made by several those fungi have been developed and extensively commercialized as Chinese cordyceps, which has caused major controversy in the industry, the public and even among some pharmaceutical scientists. Some of those fungi phylogenetically belong to cordyceps fungi, but others are phylogenetically distant from cordyceps fungi. This mini-review is to summarize the cordyceps fungi commercialized in China with a special reference to clarify Chinese cordyceps and associated fungi.

## The Chinese cordyceps industry

Chinese cordyceps is one of the best known traditional Chinese medicines, endemic to alpine regions of the Tibetan Plateau and is thought to have been discovered 2000 years ago (Zhu et al. 1998). It was officially documented for medicinal uses in the Qing dynasty (Wang 1694; Zhao 1765) to replenish the kidney and soothe the lung and was officially classified as a drug in the Chinese Pharmacopeia in 1964 (Committee of Pharmacopeia, Chinese Ministry of Health 1964). Zhang et al. (2012) suggested that *O. sinensis* should be selected as the national fungus of China.

*O. sinensis* and its ghost moth hosts are cold-adapted organisms, which resulted in many anamorphic fungi isolated in the 1970–1980s when the isolation was conducted at room temperature. At least four fungal species have been developed into pharmaceutical products, e.g. *Paecilomyces hepiali* Q.T. Chen & R.Q. Dai ex R.Q. Dai, X.M. Li, A.J. Shao, Shu F. Lin, J.L. Lan, Wei H. Chen & C.Y. Shen, *Cephalosporium sinensis* Chen (nom. nud.), *Mortierella hepiali* C.T. Chen & B. Liu and *Gliocladium roseum* Bainier (currently *Clonostachys rosea* (Link) Schroers, Samuels, Seifert & W. Gams) (Table 1, Wei et al. 2006; Zhang et al. 2013). The first three fungi were reported as new species from Chinese cordyceps (Chen et al. 1986; Jin et al. 1987; Dai et al. 1989) and the last one was reported as known species. *P. hepiali* is not the anamorph of *O. sinensis* but should affiliate to cordyceps fungus based on the current phylogenetic system of Hypocreales (Sung et al. 2007). The teleomorph of *P. hepiali* has yet to be discovered. *M. hepiali* is a species of Mortierellacea in Mortierellales, Zygomycota and apparently it does not belong to cordyceps fungi. Therefore, it should be a Chinese cordyceps–associated fungus (冬虫夏草伴生菌). *C. sinensis* was an invalid species name and was not well identified. Although most species in *Cephalosporium* have been transferred into *Acremonium*, a genus in Hypocreaceae, *C. sinensis* should be reidentified and its relationship with cordyceps fungi should be further clarified. *C. rosea* is a well-known soil-borne and mycoparasitic fungus. It phylogenetically affiliates with Bionectriaceae, Hypocreales, and its relationship with cordyceps fungi is not clear. Multigene and phylogenomic analysis indicated that the fungi in Hypocreales originated from insect association and subsequently evolved to plants and fungi associations (Nikoh and Fukatsu 2000; Spatafora et al. 2007). *C. rosea* as fungicolous fungus might be diverged from ancient insect-associated fungi and still remained the medicinal function similar to *C. sinensis* and to Chinese cordyceps. *C. rosea* and *M. hepiali* should be regarded as Chinese cordyceps–associated fungi before their relationship with cordyceps fungi being clarified.

**Table 1.** Industrialized application of fungi isolated from Chinese cordyceps.

Fungi	Relationship with Chinese cordyceps	Products	Approval number	Factory
*Hirsutella sinensis* X.J. Liu, Y.L. Guo, Y.X. Yu & W. Zeng	Anamorphic fungus of *Ophiocordyceps sinensis*	Corbrin capsule (百令胶囊)	Z10910036	East China Pharmaceutical Group Limited Co., Ltd. (中美华东制药有限公司)
		Corbrin tablets (百令片)	Z20080187	Qinghai Everest Aweto Pharmaceutical Co., Ltd.(青海珠峰虫草药业有限公司）
		Corbrin tablets (百令片)	Z20070055	Yantai Ruidong Technology Development Co., Ltd. (烟台瑞东科技发展有限公司)
		Corbrin granulation (百令颗粒)	Z20070056	Yantai Ruidong Technology Development Co., Ltd. (烟台瑞东科技发展有限公司)
*Paecilomyces hepiali* Q.T. Chen & R.Q. Dai ex R.Q. Dai, X.M. Li, A.J. Shao, Shu F. Lin, J.L. Lan, Wei H. Chen & C.Y. Shen	Cordyceps fungus associated with Chinese cordyceps	Jinshuibao capsule (金水宝胶囊)	Z10890003	Jiangxi Jiminkexin Group Co., Ltd. (江西济民可信金水宝制药有限公司)
		Jinshuibao tablet (金水宝片)	Z10910014	Jiangxi Jiminkexin Group Co., Ltd. (江西济民可信金水宝制药有限公司)
*Cephalosporium sinensis* Chen	Associated with Chinese cordyceps	Ningxinbao capsule (宁心宝胶囊)	Z13021963 etc.	110 companies such as CSPC Ouyi Pharmaceutical Co., Ltd. (石药集团欧意药业有限公司等110家企业)
		Cordyceps Cephalosporium Mycelia (虫草头孢菌粉)	H32026152 etc.	8 companies such as Jiangsu Shenhua Pharmaceutical Co., Ltd. (江苏神华药业有限公司等8家企业)
*Mortierella hepiali* C.T. Chen & B. Liu	Associated with Chinese cordyceps	Zhiling capsule (至灵胶囊)	Z14020744 etc.	5 companies such as Shanxi Datong Liqun Pharmaceutical Co., Ltd. (山西大同利群制药厂等5家企业)
		Powdered Cordyceps Mortierella Mycelia (虫草被孢菌粉)	H32026151 etc.	5 companies such as Jiangsu Shenhua Pharmaceutical Co., Ltd. (江苏神华药业有限公司等5家企业)
*Gliocladium roseum* Bainier	Associated with Chinese cordyceps	Xinganbao (心肝宝)	Z13020082	Hebei Changtian Pharmaceutical Co., Ltd. (河北长天药业有限公司)
		Cordyceps Gliocladium oral solution (虫草胶雷菌口服溶液)	H13024052	Baoding Taifu Pharmaceutical Co., Ltd. (保定孚泰药业有限公司)

### The natural Chinese cordyceps industry

The Chinese cordyceps is endemic to the Tibetan Plateau from above 3000 m altitude up to the snow line (Wang 1995; Jiang and Yao 2002), including the south-western regions of China (the Tibet Autonomous Region and Qinghai, Sichuan, Yunnan and Gansu provinces) and to some countries of the Himalayan south slope (Nepal, Bhutan and north-east India) (Shrestha et al. 2010). According to recent estimates, China accounts for more than 90% of its known production areas (Winkler 2008) and more than 95% of its annual yield (Winkler 2010). In China, the annual collection amount is about 100 t, of which Tibet and Qinghai provinces accounted for more than 80% (Ma 2010). It has been listed as ‘endangered species for protection’ in China (State Council of the People’s Republic of China 1999).

Due to the huge market demand, limited resource in nature and the failure of artificial cultivation of this fungus, the price of natural Chinese cordyceps has been rising exponentially in the last decade. In early 1970s, its price was only around 20 Renminbi (RMB)/kg, while in mid-1990s the price has increased to 5000 RMB/kg. After the outbreak of severe acute respiratory syndrome (SARS) in 2003, regarded as a panacea, Chinese cordyceps experienced skyrocketing price that exceeded around 30,000 RMB/kg and up to 100,000 RMB/kg in 2006 (Grassland Monitoring and Management Center, Ministry of Agriculture. 2010). At present, the top-quality Chinese cordyceps might be traded at 400,000 RMB/kg. It is estimated that the national annual value of natural Chinese cordyceps was over 10 billion RMB in 2004 (Yao 2004). Chinese cordyceps has become the main source of income of local farmers and herdsmen. More than 300,000 Chinese residents in the local regions rely on the collection and sale of this resource (Ma 2010). About 80% of families in the major production areas are involved in the collection of natural Chinese cordyceps, and cash income from the sales of this resource accounts for 50–80% of their total income (Ma 2010).

The artificial cultivation of fruit body of *H. sinensis* or Chinese cordyceps by infection of the ghost moth insects by *H. sinensis* has not been reported. Some ghost moths have been successfully raised in large scale and *H. sinensis* has been well fermented in factory; however, the infection rate of the ghost moths by *H. sinensis* was quite low and even infected, the fruit body production was low. The success rate of the fruit body emerging from the larvae is <1/1000 (Zhou et al. 2014). A recent study has indicated that there is high genetic diversity among *H. sinensis* isolates and a strong coevolution between ghost moth insects and *H. sinensis* (Zhang et al. 2014). The challenge is to delineate the matched *H. sinensis* strain and host insect for maximal fruit body production.

### The *Hirsutella sinensis* fermentation industry

The fall in supply and the increase in demand have stimulated interests in the search for substitutes of the natural Chinese cordyceps. As the fruit body cultivation of *H. sinensis* has not been successfully developed, the production of mycelium by fermentation is considered to be rewarding and popular. Although *P. hepiali* was isolated from Chinese cordyceps in 1982 and described as the anamorph of *O. sinensis* (Dai et al. 1989), it was invalidly published and *H. sinensis* was described and validly published later (Liu et al. 1989). *H. sinensis* is generally accepted by mycologists as the anamorph of *O. sinensis* and the notion is also supported by DNA sequence analyses by Zhao et al. (1999), Chen et al. (2001) and Liu et al. (2001, 2002) and RAPD experiments by Li et al. (2000). *H. sinensis* has been large-scale fermented and developed into medicinal products such as Corbrin capsule, tablets and granulation. Corbrin capsule is a national class I new drug, one of the state-protected traditional Chinese medicines. It can be used to nourish lung and kidney and benefit vital essence. It is effective for the treatment of cough, asthma, chronic kidney trouble, chronic hepatic trouble, functional failure, emptysis, lumbago, backache induced by weak lungs and kidneys, and chronic bronchitis (see the drug instruction of Corbrin capsule). Three companies have been approved to manufacture this medicine and the annual value has been over 1 billion RMB in 2013 in East China Pharmaceutical Group Limited Co., Ltd.

Although *Hirsutella hepiali* Chen et Shen (蝙蝠蛾被毛孢) is not validly published and is the synonym of *H. sinensis* (Jiang and Yao 2002), it has been listed in the checklist of fungi used in health food authorized by the Ministry of Health of the People’s Republic of China in 2001. At least 10 health food products with fermented mycelia as ingredient have been approved by China Food and Drug Administration.

## Industry of fungi associated with Chinese cordyceps

### Cephalosporium sinensis

*C. sinensis*, a fungus isolated from natural Chinese cordyceps, has been fermented on large scale and the mycelia have been developed to the medicine Ningxinbao capsule. There are currently over 110 companies licensed to manufacture Ningxinbao capsule. The therapeutic indications of Ningxinbao capsule include increasing rhythm of heart, improving sick sinus syndrome, benefitting the sexual ability, and improving the function of heart function and it was used for various cardiac arrhythmia, a slow-moving type of cardiac arrhythmia, and many other illness (see the drug instruction of Ningxinbao capsule). There are several health foods in the market, such as Golden Sun cordyceps oral solution and Wanji cordyceps oral solution, though it was not in the fungal checklist issued by Health Care Food by the Ministry of Health of the People’s Republic of China.

### Gliocladium roseum

*G. roseum* was isolated from Chinese cordyceps and has been developed to a medicinal product named Xinganbao capsule and cordyceps gliocladium oral solution based on the fermented mycelium. It could improve the liver function, reduce liver inflammation and fight against hepatic fibrosis (see the drug instruction of Xinganbao capsule). Hebei Changtian Pharmaceutical Co., Ltd. and Baoding Taifu Pharmaceutical Co., Ltd. are now manufacturing the products.

### Mortierella hepiali

*M. hepiali* was isolated from natural Chinese cordyceps in 1979 (Chen et al. 1986). Medicines Zhiling capsule and powdered cordyceps *Mortierella* Mycelia are derived from this fungus. It can also nourish the kidney and lung and is used for the adjuvant therapy of cough, edema and other diseases caused by lung–kidney vacuity and for the adjuvant therapy of various nephropathy, chronic bronchial asthma, chronic hepatitis and tumours (see the drug instruction of Zhiling capsule). Five companies have been approved for the production of Zhiling capsule and powdered cordyceps *Mortierella* Mycelia.

### Paecilomyces hepiali

This species was described in 1982 by scientists from the Institute of Chinese Materia Medica, China Academy of Chinese Medical Sciences based on a strain named Cs-4 isolated from natural Chinese cordyceps (Dai et al. 1989). The neotype was established recently (Wang et al. 2015) and its ITS1-5.8S-ITS2 sequence is identical with *Isaria farinose*. Cs-4 has been extensively studied in China, including industrial fermentation, chemical composition, toxicity and therapeutic functions (Ng and Wang 2005; Thakur et al. 2011). Although *P. hepiali* was isolated from Chinese cordyceps, it should be regarded as an independent cordyceps fungus other than associated fungus of Chinese cordyceps based on its phylogenetic lineage. Jinshuibao capsule, the Cs-4 fermentation product, has received approval by the National New Drug Review and Approval Committee of the Chinese Ministry of Public Health in 1989, and has been used in the clinic throughout China for replenishing the kidney, soothing the lung and beneficing pneuma. It is appropriate for patients who suffer from one or more of the following conditions: lung and kidney weakness, deficient vitality, chronic cough and asthma, fatigue and hypodynamia, insomnia and loss of memory, waist and knee adynamia, irregular menstruation, impotence and premature ejaculation, chronic bronchitis, chronic renal insufficiency, hyperlipemia, and cirrhosis (see the drug instruction of Jinshuibao capsule). There are two forms, Jinshuibao capsule and Jinshuibao tablet, produced by Jiangxi Jiminkexin Group Co., Ltd. and with an annual sale of about 2.2 billion RMB in 2014.

*P. hepiali* was also been authorized for using in health foods by the Ministry of Health of the People’s Republic of China in 2001. There are at least 18 kinds of health foods with the fermented mycelia as the major ingredient approved by China Food and Drug Administration.

### *Cordyceps militaris* industry

*C. militaris* is the type species of the newly emended genus *Cordyceps* of the family Cordycipitaceae (Sung et al. 2007), which parasitizes larva or pupa of lepidopteran insects. In China, *C. militaris* is also known as ‘North DongChongXiaCao’ and ‘BeiChongCao’ (means northern cordyceps). Recent studies have demonstrated that *C. militaris* has multiple pharmacological activities such as anti-tumour (Jin et al. 2008), anti-influenza virus (Lee et al. 2014), irradiation protective (Jeong et al. 2014) and anti-inflammatory (Smiderle et al. 2014). *C. militaris* has been increasingly viewed as a substitute for Chinese cordyceps in traditional Chinese medicine and health foods because of their similar chemical profiles and medicinal properties (Gao and Wang 2008; Yue et al. 2008; Dong et al. 2012). Unlike Chinese cordyceps fungus, *C. militaris* has a worldwide distribution but lower population density in nature. To supply the large demand for medicinal and edible purposes, large-scale fermentation of mycelium and cultivation of stromata have been extensively developed in China (Dai et al. 2007; Gu et al. 2007). Currently, the fruit bodies of this fungus have been successfully cultivated and commercialized for medicinal and health products, and even for direct consumption as edible mushroom in supermarket. In addition to the medicinal and health products from the *C. militaris* mycelia in submerged culture, the bioactive compounds (Das et al. 2010) from the fermented mycelia as pharmaceutical active ingredients, such as cordycepin, cordycepic acids, polysaccharide and carotenoids, have become a major industrial use for *C. militaris* (Holliday and Cleaver 2008).

There were two medicinal products approved by the Ministry of Health of the People’s Republic of China: *C. militaris* mycelia powder (Z20030034) and capsule (Z20030035) manufactured by Jilin Zhongsheng Pharmaceutical Co., Ltd. Their medicinal functions include invigorating the kidney, nourishing the lung, and to be effective for cough, asthma, phlegm, spontaneous perspiration, susceptible to cold, cold limbs, waist limb soft acid, fatigue, dizziness, tinnitus, etc. (see the drug instruction of *C. militaris* mycelia powder).

There are at least 36 approved health foods made from *C. militaris*. In addition, *C. militaris* can also be used as general foodstuff since it is listed as Novel Foods by the Ministry of Health of the People’s Republic of China (http://www.moh.gov.cn/publicfiles/business/htmlfiles/zwgkzt/pgg/200903/39591.htm) in 2009. Now, a huge industry based on *C. militaris* has been developed and it was estimated that the annual sale was about 3 billion in China.

### *Cordyceps guangdongensis* industry

*C. guangdongensis* was recently described as a new species from a nature reserve in southern China (Lin et al. 2008). The fruit body has been artificially cultivated and was considered safe for long-term consumption (Yan et al. 2010). Preliminary studies revealed that this fungus has multiple pharmaceutical activities such as anti-fatigue effect (Yan et al. 2013), anti-inflammatory (Yan et al. 2014), prolonging the mean lifespan and the half death time of fruit flies (Yan et al. 2011) and therapeutic effect on chronic renal failure (Yan et al. 2012). *C. guangdongensis* has been approved as Novel Foods by the Ministry of Health of the People’s Republic of China in 2013 (http://www.moh.gov.cn/zhuzhan/gonggao/201304/1b2f699e767e41248900fc3999f63bda.shtml). However, no *C. guangdongensis*-derived products have been commercialized as far as we know.

### *Issaria cicadae* industry

*I. cicadae*, also known as ‘Chan Hua’ or cicada flower, is also a famous and valuable traditional Chinese fungus. As a traditional Chinese medicine, ‘Chan-hua’ is widely used in the treatment of fever, infantile convulsion, palpitation and dizziness for thousands of years (Chen et al. 2014). Pharmacological studies have indicated that ‘Chan-hua’ possess anti-tumour (Wang et al. 2014), amelioration of renal function (Zhu et al. 2011), anti-fatigue (Wang et al. 2001), immunomodulatory (Weng et al. 2002) and sedative effects (Liu and Hu 1991).

Similar to other cordyceps species, cordyceps acid, adenosine, ergosterol and polysaccharides were isolated from this fungus (Chen et al. 2014). In addition, a sphingosine-like immunosuppressant ISP-1 (myriocin) has been isolated from the natural fruit body and fermented mycelia of *C. cicadae* (Yu et al. 2009). ISP-1 exhibited an immunosuppressive potency 10- to 100-fold greater than that of cyclosporin A, a widely used immunosuppressant, when the activity was measured in a mouse allogenic mixed lymphocyte reaction (Fujita et al. 1994). Fingolimod (FTY-720), derived from ISP-1, was approved by the US Food and Drug Administration in September 2010 as an oral drug to treat multiple sclerosis (Brinkmann et al. 2010).

Although *I. cicadae* is a cosmopolitan species in many regions of the world, its natural resources is limited to satisfy the human demand. Large-scale cultivation of its mycelia and coremium has been successful. This fungus has not been approved by authority in China either as novel food or as medicine.

## Perspective

### The Cordyceps community

It is well established that many microbes are associated with or are colonizing on natural cordyceps. Except for the 22 fungal species reported to be associated with natural Chinese cordyceps during the 1980s and 1990s (Jiang and Yao 2002), mycoflora investigation indicated the richness of fungal diversity on Chinese cordyceps based on culture-dependent and culture-independent methods (Zhang et al. 2010). Many new novel secondary metabolites have been identified from the cordyceps-colonizing fungi (Zhang et al. 2007, 2009; Guo et al. 2007; Guo, Sun, Gao, Chen, et al. 2009; Guo, Sun, Gao, Niu, et al. 2009; Ma et al. 2011). There is no doubt that the cordyceps-colonizing fungi are important resources for the discovery of novel compounds and for developing medicinal and healthy products. A coordinated effort between the industry and the scientific research community is needed to advance the whole cordyceps industry.

### Resources of cordyceps fungi

More than 500 species have been reported as cordyceps (http://www.indexfungorum.org/names/Names.asp, 2 January 2015), including 193 species of entomopathogenic fungi in *Ophiocordyceps* (MycoBank as of May 2014: http://www.mycobank.org/). Many cordyceps fungi have names of teleomorph and anamorph, and their connections need to be identified and confirmed. Roughly, three anamorph types are recognized in Ophiocordycipitaceae: *Hirsutella*-type, *Hymenostilbe*-type, and the rest as *Paraisaria*, *Tolypocladium* and *Verticillium*-type (Ban et al. 2015). Although the concept of ‘one fungus one name’ is being pushed in mycological community currently, the clarification of single name for each of cordyceps fungi is ongoing with the phylogenic analysis, which will surely result in more taxa. *C. rosea* and *C. sinensis* are not cordyceps fungi but affiliated with Hypocreales, and their medicinal function may imply that all of the fungi in Hypocreales possess the potential for medicinal tonic activities. The fungi in Hypocreales were considered to be originated from insect association and subsequently jumped to hosts of plants and fungi (Nikoh and Fukatsu 2000; Spatafora et al. 2007). The medicinal function of fungi associated with plants and fungi in Hypocreales might be remained and their medicinal and tonic functions should be extensively studied in future.

### Quality control for the industry

Although cordyceps fungi have been developed as a big industry in China and many products have been commercialized, the active chemical(s) that confer the various therapeutic claims have not been validated by vigorous studies involving chemistry, biology, pharmacology, safety assessment, and more importantly placebo-controlled clinical trials. The lack of quality control for medicinal fungi as well as other traditional Chinese medicines is a big challenge facing the modern pharmaceutical industry in China.

## Disclosure statement

No potential conflict of interest was reported by the authors.
